# Functional demonstrations of starch binding domains present in *Ostreococcus tauri* starch synthases isoforms

**DOI:** 10.1186/s13104-015-1598-6

**Published:** 2015-10-28

**Authors:** Julieta Barchiesi, Nicolás Hedin, Diego F. Gomez-Casati, Miguel A. Ballicora, María V. Busi

**Affiliations:** Centro de Estudios Fotosintéticos y Bioquímicos (CEFOBI-CONICET), Universidad Nacional de Rosario, Suipacha 531, 2000 Rosario, Argentina; Department of Chemistry and Biochemistry, Loyola University Chicago, 405 Flanner Hall, 1068 W Sheridan Road, Chicago, IL 60660 USA

**Keywords:** *Ostreococcus tauri*, Starch-binding domains, Starch synthase, Homology modeling, Adsorption assay

## Abstract

**Background:**

Starch-binding domains are key modules present in several enzymes involved in polysaccharide metabolism. These non-catalytic modules have already been described as essential for starch-binding and the catalytic activity of starch synthase III from the higher plant *Arabidopsis thaliana*. In *Ostreococcus tauri*, a unicellular green alga of the Prasinophyceae family, there are three SSIII isoforms, known as Ostta SSIII-A, SSIII-B and SSIII-C.

**Results:**

In this work, using in silico and in vitro characterization techniques, we have demonstrated that Ostta SSIII-A, SSIII-B and SSIII-C contain two, three and no starch-binding domains, respectively. Additionally, our phylogenetic analysis has indicated that OsttaSSIII-B, presenting three N-terminal SBDs, is the isoform more closely related to higher plant SSIII. Furthermore, the sequence alignment and homology modeling data gathered showed that both the main 3-D structures of all the modeled domains obtained and the main amino acid residues implicated in starch binding are well conserved in *O. tauri* SSIII starch-binding domains. In addition, adsorption assays showed that OsttaSSIII-A D2 and SSIII-B D2 domains are the two that make the greatest contribution to amylose and amylopectin binding, while OsttaSSIII-B D1 is also important for starch binding.

**Conclusions:**

The results presented here suggest that differences between OsttaSSIII-A and SSIII-B SBDs in the number of and binding of amino acid residues may produce differential affinities for each isoform to polysaccharides. Increasing the knowledge about SBDs may lead to their employment in biomedical and industrial applications.

**Electronic supplementary material:**

The online version of this article (doi:10.1186/s13104-015-1598-6) contains supplementary material, which is available to authorized users.

## Background

The generic term carbohydrate binding module (CBM) refers to a contiguous amino acid sequence within a carbohydrate-active enzyme with a distinctive fold presenting carbohydrate-binding activity [1, 2]. Enzymes possessing CBMs share a common modular organization, including a catalytic domain typical for each enzyme and one or more CBMs connected by a loosely-structured chain. CBMs can be found in the middle, N- or C-terminal positions of the polypeptide chain [3]. They are currently grouped in 71 different families based on their amino acid sequences, substrate binding specificities, location in protein and structures (see http://www.cazy.org/fam/acc_CBM.html) [1]. CBMs from different families are structurally similar. Their ability to bind carbohydrates can be attributed, at least partially, to several aromatic residues which assemble into a hydrophobic surface.

Among CBMs, we can highlight the Starch Binding Domains (SBD), which have acquired the evolutionary advantage of being capable of disrupting the surface of their substrate [4–6]. These domains are distributed in twelve CBM families; 20, 21, 25, 26, 34, 41, 45, 48, 53, 58, 68 and 69 [7–10]. The CBM53 family currently has about 96 entries in the CAZy database, representing high phylogenetic diversity, as CBM53 s are found in archaea, bacteria, and eukaryotes. The characterized entries correspond to starch synthase III (SSIII) enzymes belonging to higher plant species (*Arabidopsis thaliana*, *Zea mays*, *Solanum tuberosum*, *Phaseolus vulgaris*, *Vignia unguiculata* and *Oryza sativa*) and *Chlamydomonas reinhardtii*. On the other hand, *Ostreococcus tauri* SSIII-A and SSIII-B sequences are included in the CBM53 non-characterized entries [11].

Three-dimensional structures are available for nine of the twelve families, except for those classified into CBM45 and CBM53 families. In general, SBD modules comprise 90–130 residues [12–14], and normally maintain functionality in isolated form [6, 7]. Despite their low sequence similarity, the nine remaining families do share very similar folds, composed by several β-strands forming an open-sided distorted β-barrel [6, 7, 15, 16].

An outstanding example of the importance and functionality of an SBD in a plant biosynthetic enzyme is the case of the SSIII isoform from *A. thaliana* (ArathSSIII), a key regulatory enzyme in starch metabolism, with three SBDs located in tandem in its N-terminal domain, belonging to the CBM53 family [17, 18]. These SBDs, named D1, D2 and D3, have a known regulatory role on ArathSSIII, presenting starch binding activity and also being involved in the modulation of the catalytic properties of the enzyme [17–20].

In the last few years, several nuclear and organellar algae genomes have been sequenced. Some nuclear sequenced genomes from green algae include those from *O. tauri* [21, 22], *O. lucimarinus* [23], *C. reinhardtii* [24], *Micromonaspusilla* [25], *Chlorella variabilis* [26] and *Volvox carteri* [27], among others. *C. reinhardtii* and *O. tauri* genomes are the best characterized, as documented in numerous publications [21–24]. Although *C. reinhardtii* has been a model organism for several decades [28], *O. tauri* has lately gained importance since its first description in 1994 [29].

*O. tauri* is a picophytoplanktonic species that belongs to the Prasinophyceae, a group of green algae which is thought to have diverged very early from the ancestor of all chloroplast-containing green plants and algae [21, 22]. Although *O. tauri* constitutes the tiniest eukaryotic cell known and presents the smallest genome of a eukaryotic photosynthetic organism described to date, it contains more SSIII-like genes (three copies) than any other algae and plant species (e.g., *A. thaliana* presents only one copy) [30]. Thus, the genome of *O. tauri* encodes three SSIIIs, SSIII-A, SSIII-B and SSIII-C, all of which remain uncharacterized to date. The conservation throughout evolution of the three SSIII isoforms, and the absence of SSIV, could be related to the presence of a single starch granule in this alga, which presents similar composition than higher plant starch, but a particular partitioning and propagation mechanism [31].

In summary, this work represents the first characterization of a SBD in algae and based on the results obtained from the in silico analysis, together with the binding profile obtained from the polysaccharide adsorption assays, we postulate that *O. tauri* SSIII-A and SSIII-B proteins possess two and three SBD domains, respectively. In addition, our binding assay results are evidence that both *O. tauri* SSIII isoforms have different polysaccharide binding specificities.

## Results and discussion

### Sequence alignment and phylogenetic analysis of the Ostta SSIII SBD domains

Similarity searches using PSI-Blast [32] retrieved numerous sequences whose GeneBank codes are shown in Additional file 1: Table S1. When sequences corresponding to OsttaSSIII-A and OsttaSSIII-B N-terminal regions, and OsttaSSIII-C entire sequence were used as input, we found sequence similarities with respect to the SSIII from different plants and algae displaying considerably low E-values. In addition, OsttaSSIII-A was very similar (E = 6 × 10^−5^) to a hypothetical protein from *Desulfosporosinus meridiei*, a bacterium isolated from groundwater contaminated with aromatic compounds derived from fuels [33]. In turn, OsttaSSIII-C also presents high sequence similarity with a hypothetical protein from *Parachlamydia acanthamoebae* str. Hall’s *coccus* and also with glycogen synthases from *Candidatus Protochlamydia amoebophila* UWE25 and from cyanobacteriae *Anabaena variabilis* ATCC 29413, *Synechococcus* sp. JA-3-3Ab and *Fischerella* sp. JSC-11.

**Fig. 1:**
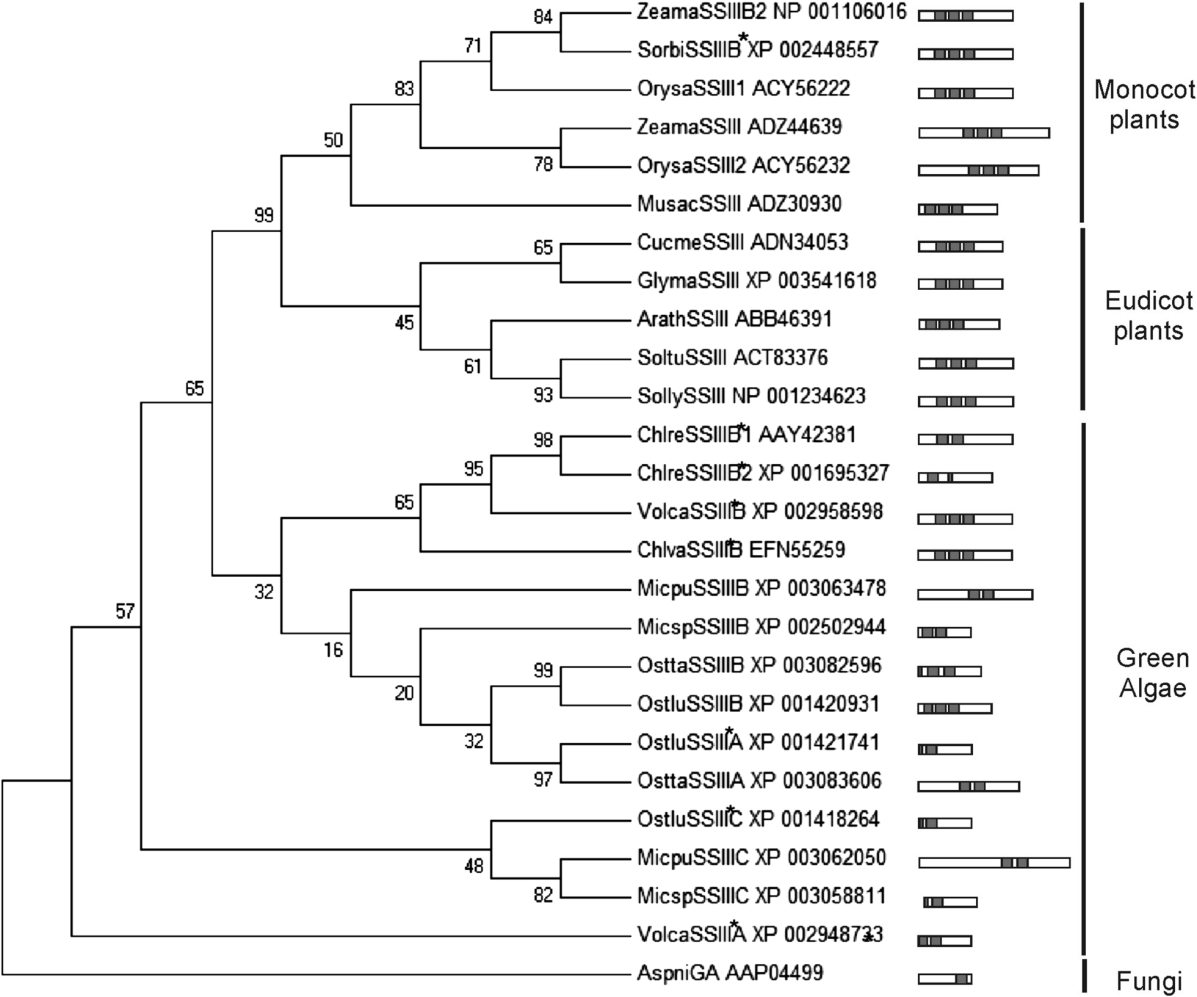
Phylogenetic tree of SSIII SBD regions. The tree is based on the alignment of SBD regions from SSIII amino acid sequences obtained with PSI-Blast (shown in Additional Table 1). It was built by Maximum Parsimony method in MEGA 6.06 version [57] and it is rooted to *A. niger* amyloglucosidase (AspniGA) CBM20 (used as outgroup sequence). Phylogenies were determined by Bootstrap Analysis of 500 replicates in MEGA 6.06 version [59]. Branch lengths are proportional to distances. Bootstrap values are shown above branches Abbreviations of the source proteins: Ostlu, *O. lucimarinus*; Micsp, *Micromonas* sp. RCC299; Ostta, *O. tauri*; Micpu, *Micromonas pusilla* CCMP1545, Chlre, *Chlamydomonas reinhardtii*; Volca, *Volvox carteri*; Chlva, *Chlorella variabilis,* Zeama, *Zea mays*; Sorbi, *Sorghum bicolor*; Orysa, *Oryza sativa*; Musac, *Musa acuminata*; Soltu, *Solanum tuberosum*; Arath, *Arabidopsis thaliana*; Cucme, *Cucumis melo;* Glyma*, Glycine max.* *Asterisk* indicate hypothetical proteins names assigned by *O. tauri* SSIIIs homology. The schematic representation of SBD number and position (in *grey color*) from each SSIII protein is included on the *right side* of the figure

The alignment of the amino acid sequences from OsttaSSIII-A and OsttaSSIII-B SBD regions with homologous sequences identified with PSI-Blast, allowed us to construct a phylogenetic tree for these regions (Fig. 1). The evolutionary relationships deduced from this analysis suggest that OsttaSSIII-A and OsttaSSIII-B SBDs containing regions are most closely related to other alga SSIII proteins rather than to higher plants SSIII, including *A. thaliana* (Fig. 1).

**Fig. 2:**
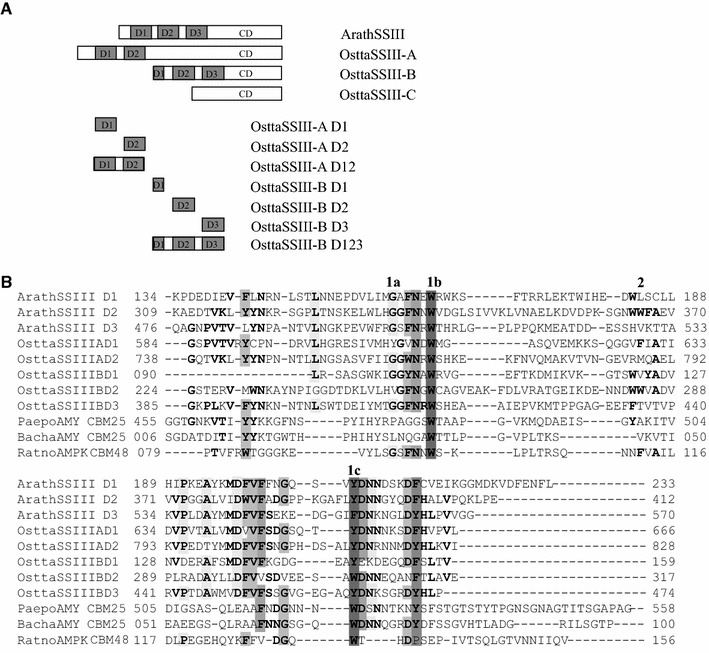
**A** Schematic representation of ArathSSIII, OsttaSSIIIs and OsttaSSIII recombinant SBDs. CD, catalytic domain. **B** Alignment between OsttaSSIII-A D1 and D2, OsttaSSIII-B D1, D2 and D3, ArathSSIII D1, D2, and D3, and RatnoAMPK β-subunit GBD (CBM48), BachaAMY CBM25 and PaepoAMY CBM25 used as the template for homology modeling. 1 and 2 above the alignment indicate binding-site 1 and 2 characterized in *A. thaliana*. 1a, 1b and 1c indicate G335, W340 and Y394, and 2 W366 (all correspond to *A. thaliana* numbering, [20]), respectively. Other conserved positions are shown in *bold* and four levels of *gray* represents 100, 90, 82, 78 and 55 % of conservation

Subsequently, using InterPro [34] and CD-search servers [35], a different number of SBDs were detected in OsttaSSIIIs N-terminal and central protein regions with significantly low E-values (threshold 1 × 10^−4^). Three domains were detected in OsttaSSIII-B (D1, D2, and D3, named in order from N-terminal to C-terminal), two in OsttaSSIII-A (D1 and D2) and none in OsttaSSIII-C (Fig. 2A). The five domains share 19–40 % identity (CLUSTALW [36]). All of them have typical 90–130 aa SBD sizes [13, 14], with the exception of OsttaSSIII-B D1, which is 59 aa long. The analysis of SSIII N-terminal regions from other green algae reveals that *O. lucimarinus*, *M. pusilla* CCMP1545, *M.* sp. RCC299 and *C. reinhardtii* show variable numbers of SBDs in their SSIII isoforms, with some of them exhibiting a reduced size (between 37 and 62 aa) (Fig. 1). These aspects seem to be exclusive of green algae SSIII SBDs, given the fact that all the land plant species with multiple SSIII isoforms analyzed to date present three SBDs with definite size conservation.

Despite the fact that there is no structural information available for the N-terminal D2 domain from ArathSSIII, it is by far one of the best characterized starch binding domains studied. We have previously reported that ArathSSIII D2 contains two binding sites which comprise amino acid residues Y394 (binding site 1) and W366 (binding site 2) acting cooperatively in the binding process with domain D1, while residues G336 and W340 have minor contributions [20]. The alignment of OsttaSSIII and ArathSSIII SBDs sequences shows a high degree of similarity, indicating that these domains are well conserved (Fig. 2B). OsttaSSIII-B D1 and OsttaSSIII-B D2 present higher sequence identity with ArathSSIII D2 (25 and 21.2 % identity, respectively), than what is observed with ArathSSIII D1 and D3. Meanwhile, OsttaSSIII-A D1, OsttaSSIII-A D2 and OsttaSSIII-B D3 amino acid sequences are more similar to ArathSSIII D3 (29.5, 40 and 43 % identity, respectively), than to ArathSSIII D1 and D2.

Furthermore, the amino acid residues involved in ArathSSIII D2 starch binding are highly conserved in OsttaSSIII SBDs. There is high conservation of residues G335, W340 and Y394 [the first one is exchanged by a tyrosine residue in OsttaSSIII-A D1 and by a valine residue in OsttaSSIII-B D2, and the last one is exchanged by a tryptophan residue in OsttaSSIII-B D2 (Fig. 2B)]. On the other hand, W366, proposed to be essential in ArathSSIII D2 binding site 2 for the binding to amylose, amylopectin and starch, is only conserved in OsttaSSIII-B D2. This residue was replaced by a phenylalanine in OsttaSSIII-A D1, and by a methionine, a valine and a threonine in OsttaSSIII-A D2, OsttaSSIII-B D1 and OsttaSSIII-B D3, respectively. Our results suggest that the second polysaccharide binding site seems to be exclusive of OsttaSSIII-A D1 and OsttaSSIII-B D2 domains. However, the neighboring residue W365 is conserved in OsttaSSIII-B D1 and OsttaSSIII-B D2. Further investigation is necessary to analyze its possible role in polysaccharide binding.

### Molecular model of the OsttaSSIII SBDs

**Fig. 3:**
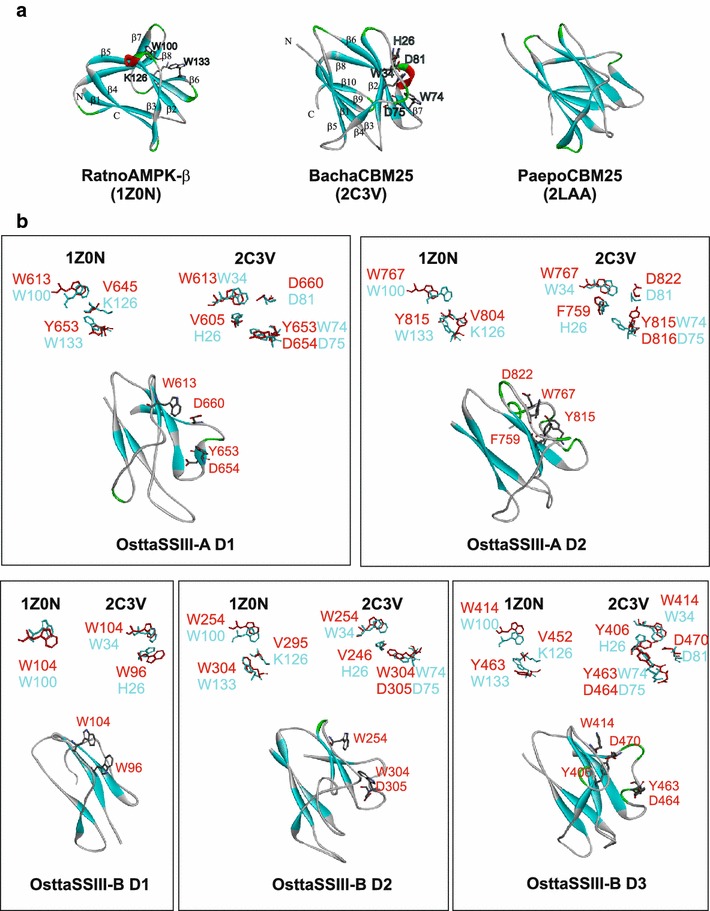
Homology modeling of OsttaSSIIIs SBDs. **a** Structural model of *Rattus norvegicus* AMPK β-subunit GBD (RatnoAMPK-β, PDB code: 1Z0N), *Bacillus halodurans* amylase CBM25 (BachaCBM25, PDB code: 2C3V) and *Paenibacillus polymyxa* amylase CBM25 (PaepoCBM25, PDB code: 2LAA); binding residues are shown. **b** Proposed models for OsttaSSIII-A D1, OsttaSSIII-A D2, OsttaSSIII-B D1, OsttaSSIII-B D2 and OsttaSSIII-B D3; putative binding residues are shown. Superimposition of OsttaSSIII-A D1, OsttaSSIII-A D2, OsttaSSIII-B D1, OsttaSSIII-B D2 and OsttaSSIII-B D3 binding residues (*red*) and 1Z0N or 2C3V (*cyan*) binding residues, is shown next to each model

The homology models of the OsttaSSIII SBDs based on CBM25-1 of beta/alpha-amylase from *Paenibacillus polymyxa* (PDB code: 2LAA), CBM25 from *Bacillus halodurans* amylase (PDB code: 2C3V) and *Rattus novergicus* glycogen-binding (GBD) domain of the AMP-activated protein (PDB code: 1Z0N) [37–39], are shown in Fig. 3b. To obtain the homology models, we used the three templates in parallel (Fig. 3a), using the best part of each template with the Modeller program. Regardless of the low identity with these templates (10–23 % for OsttaSSIII-A D1, OsttaSSIII-A D2, OsttaSSIII-B D2 and OsttaSSIII-B D3), the four OsttaSSIII SBDs were predicted by threading to have a similar β-sandwich fold. The confidence of the prediction using GenTHREADER [40, 41] was high, with the *p* value lower than 10^−3^ for 2C3V and 2LAA templates. Although 1Z0N template p-value was around 10^−2^, it was also included because of the high score obtained for it in a previous fold class assignation with @TOME V2 [42]. On the other hand, OsttaSSIII-B D1 identity with the templates was inferior (9–11 %), mainly due to its short amino acid sequence, and the confidence of the prediction using GenTHREADER was low. However, OsttaSSIII-B D1 model was also generated with the same three templates (PDB: 1Z0N, 2C3V and 2LAA) as a first approximation to estimate a short starch binding domain fold.

It is important to note that the templates may have similar roles to the domains modeled. AMPK is an AMP-activated protein kinase responsible for the coordination of cellular metabolism in response to energy demands and many other stimuli. AMPK-β subunit acts as a platform for the α-catalytic and γ-regulatory subunits and targets the AMPK heterotrimer to glycogen [39, 43, 44]. The AMPK-β glycogen binding domain belongs to the CBM48 family, and presents a 24–25 % sequence identity with OsttaSSIII-A D1, OsttaSSIII-A D2, OsttaSSIII-B D2 and OsttaSSIII-B D3. The family is mainly represented by CBMs appended to GH13 subfamily [45] and the beta subunit (glycogen-binding) of mammalian AMP-activated protein kinases (AMPK) [39]. It is worth mentioning that there is a clear evolutionary relatedness of the CBM48, CBM53 and CBM20 families, which suggests the existence of a common clan hosting most of the known SBDs [7]. Moreover, the alignment of the amino acid sequences of CBM20, CBM21, CBM48 and CBM53 illustrates the close evolutionary relationship of the four CBM families and reveals only subtle differences in the polysaccharide-binding sites, showing a high degree of conservation [7].

Instead, *P. polymyxa* amylase and *B. halodurans* alpha/beta amylase, both extracellular bacterial starch degrading enzymes, present starch binding domains belonging to CBM25 family. The modules from this family are frequently linked to amylolytic enzymes from the family GH13, but there are CBM25 examples found in families GH14, GH31 and GH119 [9, 46]. With regard to *B. halodurans* amylase, it contains both CBM26 and CBM25. Each SBD binds starch individually, though the affinity for both modules together was 50 times higher [9, 37].

Based on the results obtained with the validation tools Verify 3D and Rampage, we are able to state that our models exhibit good quality, but most importantly, certainly suggests a strong structural similarity among these domains and their templates considering the level of sequence identity observed.

All the SDB models created show a similar fold to that described for RatnoAMPK-β GBD and BachaAMY CBM25 with a compact β sandwich structure composed of two antiparallel β-sheets (Fig. 3). One β-sheet from AMPK-β GBD is formed by strands β1 and β4, and the other β-sheet contains strands β2, β3, β5 and β6 plus a β-hairpin β7-β8. The AMPK-β GBD structure presents a carbohydrate binding pocket with residues W100, W133, K126, L146, G147, T148 and N150 (1Z0N numbering) involved in polysaccharide binding [39, 43, 47]. Residues W100 and W133 correspond to W340 and Y394 (ArathSSIII D2 numbering), implicated in starch binding in ArathSSIII D2 [20]. The BachaAMY CBM25 structure in complex with maltotetraose shows a polysaccharide binding site formed by the residues W34, W74, H26, D81 and N76 (2C3V numbering). BachaAMY CBM25 has only one main maltooligosaccharide binding site [37]. Residues W34 and W74 correspond to W340 and Y394 involved in starch binding in ArathSSIII D2, as mentioned above [20].

Despite the definite conservation of the main 3-D structures of all the modeled domains, there are some minor differences. None of them have the β7-β8 hairpin loop present in RatnoAMPK-β GBD at the C-terminal end. OsttaSSIII-A D1 and OsttaSSIII-A D2 also lack the β3 strand. Absence of the β3 strand is a common feature of CGTases [48] and maltogenic α-amylases [7, 49]. Besides, the β-hairpin β7-β8 is missing in previously identified folding of the E domain of a cyclodextrin glycosyltransferase and the C-terminal domain of a β-amylase from both *Bacillus cereus* and *Aspergillus niger* glucoamylases [39]. Further investigation is necessary to analyze the implications of the differences among the OsttaSSIII SBDs structures that ultimately affect their polysaccharide binding specificities and biological functions.

A comparison of the RatnoAMPK-β GBD subunit, BachaAMY CBM25 and PaepoAMY CBM25 with those from OsttaSSIII SBDs showed a 100 % conservation of the W100/W34 residue (1Z0N/2C3V numbering) in all sequences (Fig. 2B). Besides, these residues have a conserved position in the five models proposed (Fig. 3b). Instead, the W133/W74 residue (1Z0N/2C3V numbering) is conserved in OsttaSSIII-B D2 sequences, but is replaced by tyrosine in the other four analyzed sequences. Moreover, these residues do present a similar spatial location in the models, with exception of OsttaSSIII-B D1 which lacks an aromatic amino acid in this spatial region. Regarding the spatial position of the basic residue K126 (1Z0N numbering), it was replaced by a valine residue in OsttaSSIII-A D1, -A D2, -B D2 and -B D3, and is absent in the OsttaSSIII-B D1 model structure. This particular amino acid replacement in the binding site could be a consequence of architectural disparities between starch and glycogen. On the other hand, a conservation of an aromatic or aliphatic side chain suggests the importance of a possible hydrophobic interaction at this point.

We also noted a superposition of the aspartic residue at D75 (2C3V PDB numbering) in the OsttaSSIII-A D1, -A D2, -B D2 and -B D3 models. Besides, the spatial location of residue D81 (2C3V numbering) is conserved in OsttaSSIII-A D1, -A D2 and -B D3, and it is replaced by phenylalanine in OsttaSSIII-B D2. On the other hand, the H26 residue (2C3V numbering) is replaced by aromatic residues such as phenylalanine (in OsttaSSIII-A D2), tryptophan (in OsttaSSIII-B D1) and tyrosine (in OsttaSSIII-B D3). The role that these residues might play in the starch binding activity of these domains is analyzed below.

### OsttaSSIII SBDs polysaccharide binding assays

Carbohydrate binding modules are frequently associated with carbohydrate active enzymes, specifically those that are active on polysaccharides. They have several functions: align the substrates with the reaction centers, disrupt the surface of the substrates and provide support for macromolecular aggregates. However, the presence of CBMs is not universal and is in fact rare among some families of enzymes [50]. There are examples of enzymes that are active on polysaccharides and have no CBMs in structure. Their catalytic domains contain residues that are responsible for the binding of the carbohydrate to the enzyme, but distant from the active site. These residues form a surface site or a secondary binding surface (SBS). The functions of the SBSs are not limited to the orientation of the enzyme to the substrate, but also include the substrate guidance to the active site and allosteric site, altering the specificity of the enzyme. Within families of GH13 and GH77 about 45 enzymes with one SBS were identified. SBSs and CBMs usually co-occur in enzymes and they probably have complementary functions [50].

The different number of SBDs in OsttaSSIII isoforms may be indicative of different functional characteristics. The binding efficiency of individual or tandem recombinant purified OsttaSIII SBDs (see Fig. 2A) to insoluble polysaccharides was evaluated. We analyzed their ability to bind starch, amylose and amylopectin in co-sedimentation and adsorption assays.

As shown in Additional file 2: Figure S1, all individual OsttaSSIII SBD were found in the pellet with amylose and amylopectin at different levels, except OsttaSIII-A D1. This domain lacks the amino acid residues equivalents to G335 and W365, which are involved in polysaccharide binding in ArathSSIII D2 (Fig. 2) [20]. *In tandem* OsttaSIII-A D12 bound remarkably to amylose, probably as a result of different amino acids exposed to the polysaccharide and also by the presence of a cooperative effect between OsttaSIII-A D1 and OsttaSIII-A D2. On the other hand, OsttaSIII-A D12 did not bind to amylopectin. Its higher molecular mass could be a steric impediment against this branched substrate. Under the assayed conditions, starch binding capacity was observed only in OsttaSIII-B D1 and OsttaSIII-B D123 (Additional file 2: Figure S1). Consistently, when *Aspergillus niger* amyloglucosidase was used as a positive binding control, it showed higher co-sedimentation towards amylose rather than starch.

**Fig. 4:**
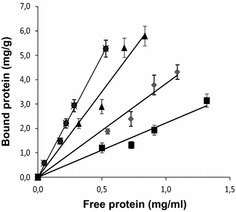
Adsorption of purified OsttaSSIII SBD proteins to polysaccharides: OsttaSSIII-A D12 with amylose (*filled circle*),OsttaSSIII-B D123 with amylose (*filled triangle*),OsttaSSIII-B D123 with amylopectin (*filled diamond*), and OsttaSSIII-B D123 with starch (*filled square*). Linear adsorption isotherms indicate the apparent equilibrium distribution of OsttaSSIII SBD proteins between the solid (bound protein) and liquid phase (free protein) at different protein concentrations. *K*_ad_ (milliliters per gram of polysaccharide) values represent the slopes of each isotherm

To further characterize the polysaccharide binding affinity, we determined the adsorption constant (*K*_ad_) in an adsorption assay, at different protein concentration as was previously described [19]. Figure 4 shows the adsorption isotherms for the binding of OsttaSSIII-A D12 and OsttaSSIII-B D123 to starch, amylose and amylopectin. Table 1 presents OsttaSSIII SBDs determined *K*_ad_ values [19].

**Table 1:** Adsorption constants for OsttaSSIII SBD recombinant proteins

Isoform	*K*_ad_ (mL/g)		
	Starch	Amylose	Amylopectin
OsttaSSIII-A D1	ND	ND	ND
OsttaSSIII-A D2	ND	7.32 ± 0.53	7.50 ± 0.65
OsttaSSIII-A D12	ND	9.95 ± 0.82	ND
OsttaSSIII-B D1	3.70 ± 0.43	7.13 ± 0.61	3.12 ± 0.34
OsttaSSIII-B D2	ND	7.23 ± 0.42	8.22 ± 0.59
OsttaSSIII-B D3	ND	1.84 ± 0.20	3.12 ± 0.28
OsttaSSIII-B D123	2.22 ± 0.21	7.02 ± 0.44	3.84 ± 0.31

*ND* no determined

Consistently with co-sedimentation assay, all recombinants OsttaSSIII SBD showed amylose and amylopectin affinity, except OsttaSIII-A D1 (Table 1). *In tandem* OsttaSIII-A D12 presents higher amylose affinity, and starch binding capacity was observed only in OsttaSIII-B D1 and OsttaSIII-B D123, and in lower levels with respect to amylose.

The OsttaSIII-B D1 domain binds to all the polysaccharides assayed, despite its small size. This 59 amino acid residue module conserves all starch binding amino acids described for ArathSSIII D2, with the exception of W366; however, W365 and/or Y367 could compensate for its absence, probably as an alternative binding site. As already mentioned, it is important to highlight that OsttaSSIII-B D1 presents higher sequence identity with ArathSSIII D2 than what is observed with ArathSSIII D1 and D3. In addition, the aromatic residue W96 (OsttaSSIII D1 numbering) is in a similar spatial position than the residue H26 (2C3V numbering) suggesting a role in polysaccharide binding (Fig. 3b), however, this remains to be determined. These results suggest that the different SBD organization in OsttaSIII isoforms could provide precise polysaccharide affinities, reinforcing the evolutionary conservation of the isoforms. It stands to reason that additional investigation will be required to test this premise.

In summary, we have found that *O. tauri* SSIII-A and SSIII-B isoforms present two and three SBD, respectively. This inference is based on fold class assignment results, sequence alignment data, the analysis of the conservation of specific residues implicated in starch binding and the results obtained from the polysaccharide binding assays. The statistical evaluation of the models obtained using independent methods, as Rampage evaluation criteria and the Verify-3D structure evaluation server, gives further support to the assignment of the OsttaSSIII SBDs folds to CBM family 25, 48 and 53 such as ArathSSIII D1, D2, and D3. The construction of a phylogenetic tree allowed us to determine that OsttaSSIII-A and OsttaSSIII-B SBD containing regions are evolutionarily closer to other green algae SSIIIs rather than the enzymes from higher plants. Additionally, we observed variable SBD sizes and amounts in green algae SSIII enzymes. Therefore, our results suggests that *O. tauri* possesses three SSIII isoforms, one similar to higher plants SSIII, presenting three N-terminal SBDs (OsttaSSIII-B) and the other two isoforms, one with two central SBDs (OsttaSSIII-A) and the third lacking SBDs (OsttaSSIII-C). The biological significance of the last two should be further studied.

Most of the residues responsible for binding to glycogen in *Agrobacterium tumefaciens* GlgA are conserved in the catalytic domain of ArathSSIII [51] and OsttaSSIII (Additional file 3: Figure S2). Four of seven residues analyzed and listed in Busi et al. 2008 [51] are present in OsttaSSIII-C (E25, Y112, D146 and R270, OsttaSSIII-C numbering) and may constitute an SBS. We could infer that the difference in the mechanism of action of the three OsttaSSIII proteins was mainly due to the combined action of SBDs and SBSs in OsttaSSIII-A and OsttaSSIII-B and only SBSs in OsttaSSIII-C.

SBSs and SBDs have been studied in great detail beginning a few years ago. Researchers have developed strategies for the identification and characterization of these sites, using techniques that measure their binding properties as well as looking at the influence on enzymatic activity of altering these sites through mutagenesis. Our group has recently published the optimization of the kinetic parameters of a GlgA from bacteria by adding one, two or three plant SBDs which have the capacity to bind to polysaccharides. Our results also showed that the chimeric enzyme has an increased capacity to synthesize glycogen in vivo [52].

This growing interest to study SBDs may eventually lead to applications involving them in industrial and biomedical settings [53–55]. These modules provide an interesting way to regulate enzymatic activity without altering the structure of the active site of the enzyme. Due to OsttaSSIII SBDs ability to bind to starch, amylose and amylopectin, we propose the use of these modules as biotechnological tools for modifying the quality and/or amount of starch in plants and algae. In conclusion, our results constitute an important cluster of information concerning evolutionary and structure–function aspects of SBD domains.

## Methods

### Homology modeling and sequence alignment

The sequence similarity searches of OsttaSSIII-A (Genbank code: XP_003083606, 1-843 aa residues) N-terminal region, OsttaSSIII-B (Genbank code: XP_003082596, 1-386 aa residues) N-terminal region and OsttaSSIII-C (Genbank: XP_003079955) whole amino acid sequence were performed with PSI-BLAST [32] with the default parameters (inclusion threshold 0.005) until convergence, using no redundant databases. Sequences with significant matches to the query sequence (threshold expectation value (“E value”) below 1 × 10^−4^) were retrieved and aligned with the program CLUSTALW [36]. Alignment of the OsttaSSIII-B N-terminal amino acid sequences with homologous sequences were performed by using the Unipro UGENE v.1.10.4 program [56] with default parameters. Alignment of the OsttaSSIII, ArathSSIII, AgrtuGS and EsccoGS catalytic domain (CD) amino acid sequences were performed by using the Unipro UGENE v.1.10.4 program [56] with default parameters. Phylogenetic SSIII SBD regions tree was built by Maximum Parsimony method in MEGA 6.06 version [57]. The tree was rooted by *A. niger* amyloglucosidase CBM20. Phylogenies were determined by Bootstrap Analysis of 500 replicates in MEGA 6.06 version.

We have used two approaches to detect domains in OsttaSSIIIs N-terminal region: the CD-search server [35] and the InterPRO resource [34]. SBDs were aligned with the program CLUSTALW [36] and manually improved with the program Bioedit [58].

Threading and fold recognition were achieved using the program GenTHREADER [40, 41]. Homology modeling of OsttaSSIIIs SBD domains was performed using the program Modeller 9.13 [59]. Each structure was modeled using three templates; CBM25-1 of beta/alpha-amylase from *Paenibacillus polymyxa* (PDB code 2LAA), CBM25 from *Bacillus halodurans* amylase (PDB code 2C3V) and *Rattus novergicus* glycogen-binding domain of the AMP-activated protein complexed with beta-cyclodextrin (PDB code 1Z0N) [37–39, 47]. Alignment with the templates was based on homology and secondary structure. The reliability of the model was evaluated using the programs Rampage (a structure validation tool for the assessment of the Ramachandran plot of proteins) [60] and Verify3D [61]. Superimposition of structures was performed using the MultiProt server [62].

### Cloning, expression and purification of OsttaSSIII SBD

Starch binding domains OsttaSSIII-A D1, D2 and tandem D12 (**Ot16g01560**) were cloned from *O. tauri* genomic DNA (kindly provide for Dr. Evelyne Derelle) into *EcoR*I and *Hind*III sites of pRSET-B (Invitrogen CA, USA). Starch binding domains OsttaSSIII-B D1, D2, D3 and tandem D123 were cloned from *O. tauri* genomic DNA (**Ot13g01250**) into *Kpn*I and *Hind*III sites of pRSET-C (Invitrogen CA, USA), using standard molecular biology procedures and the primers: AD1 Fw, CCAGAATTCGGATCACCCGTC; AD1 Rev, CGAAAGCTTGAGAACGGGAACATG; AD2 Fw, TCGAATTCGGGCAGACTGTCAAG; AD2 Rev, CGAAAGCTTTGAGGTGATAATCC; BD1 Fw, TGGGTACCCATCGGTGGATACAACGCG, BD1 Rev, GCGAAGCTTCGAACTAGACAGTC; BD2 Fw, GGGGTACCCGGGAGCACGGAGCG; BD2 Rev, CTTAAGCTTTTACTCCACTGCGAGCG; BD3 Fw, CCGGTACCTGGCAAACCGCTCAAG; and BD3 Rev, GCGAAGCTTCCTCGCTAGGGGAG. These expression vectors were transformed in *Escherichia coli* BL21 (DE3) pLysS strain. Cells were grown at 37 °C for 3 h, 1 mM IPTG was added and incubated at 30 °C for at least 4 h. Cells were harvested by centrifugation at 5000×*g* for 15 min at 4 °C. Each pellet was suspended in buffer containing 20 mM Tris–HCl (pH 7.5). Cells were disrupted by sonication and centrifuged at 12000×*g* for 15 min at 4 °C. The homogenates were loaded onto a HiTrap chelating HP column (GE Healthcare BioSciences, Uppsala, Sweden) equilibrated with binding buffer [20 mM Tris–HCl (pH 7.5) and 20 mM imidazole]. The column was washed with 10–15 volumes of binding buffer, and each protein was eluted using a linear gradient of binding buffer and elution buffer [20 mM Tris–HCl (pH 7.5) and 20–500 mM imidazole] [18]. The presence of the SBD in elution fractions was monitored by SDS–PAGE analysis. Obtained SBD sizes: OsttaSSIII-A D1 16 kDa, OsttaSSIII-A D2 15 kDa, tandem OsttaSSIII-A D12 34 kDa, OsttaSSIII-B D1 8 kDa, OsttaSSIII-B D2 11 kDa, OsttaSSIII-B D 3 10 kDa, tandem OsttaSSIII-B D123 48 kDa. The protein containing fractions were concentrated to >1 mg/mL using Vivaspin 6 3000 MWCO concentrators (GE Healthcare BioSciences, Uppsala, Sweden), desalted and stored at 4 °C until they were used.

### Polysaccharide-binding assays

Purified SBD above described (approximately 20 μg each) were mixed with starch (Fluka-85649, St. Louis, MO, USA), amylose (Fluka-10130, St. Louis, MO, USA) or amylopectin (Fluka10118, St. Louis, MO, USA) in 20 mM Tris–HCl (pH 7.5), at a final polysaccharide concentrations of 10 % (w/v). A control was done without polysaccharide. Binding was done at room temperature by orbital mixing for 30 min. Polysaccharides were obtained by centrifugation at 12000×*g* for 5 min, and the supernatant removed and boiled in SDS loading buffer. The pellets were washed three times with 100 µL 20 mM Tris–HCl (pH 7.5) by gentle vortexing and centrifugation, and then they were boiled in SDS loading buffer in a final volume equal to the supernatants for SDS–PAGE [63]. Protein levels were determined by densitometry of SDS-PAGE gels that had been stained with 0.25 % Coomassie Blue R-250 in 45 % v/v methanol, 10 % v/v acetic acid, then destained in 25 % v/v methanol, 7 % v/v acetic acid, using a scanner and Adobe Photoshop (Adobe Systems Inc., Mountain View, CA). *Arabidopsis thaliana* frataxin was used as negative control [64].

The adsorption constant (Kad) of SBD was measured as described previously with minor modifications [18, 19]. Purified recombinant SBD proteins (final concentration of 0–80 μM) were mixed with starch (Fluka-85649, St. Louis, MO, USA), amylose (Fluka-10130, St. Louis, MO, USA) or amylopectin (Fluka10118, St. Louis, MO, USA) in 20 mM Tris–HCl (pH 7.5), at a final polysaccharide concentrations of 10 % (w/v). A control was done without polysaccharide. Binding was done at room temperature by orbital mixing for 30 min and centrifuged at 12000×*g* for 5 min at 4 °C. To calculate the amount of bound protein, the amount of protein in the supernatant was subtracted from the total protein added to the mixture. The protein concentration was determined by Lowry method. The adsorption constant (Kad, in milliliters per gram of polysaccharide) was determined from the slope as previously reported [18, 19].

## Additional files

10.1186/s13104-015-1598-6 Sequences Retrieved from Similarity Searches.
10.1186/s13104-015-1598-6 OsttaSSIII SBD-polysaccharide co-sedimentation assay. **A.** OsttaSSIIIs SBDs were incubated with starch, amylopectin, or amylose and examined for their ability to co-sediment. Then, they were localized to the supernatant (S) or pellet (P) fraction in SDS-PAGE, as described in Methods section. **B.** Densitometric quantifications of OsttaSSIII SBD-polysaccharide co-sedimentation assay presented in A. Polysaccharide bound or free protein levels are shown. The density for supernatant fraction of buffer incubated samples (control) was set to 100 % for each experiment, and density for pellet fraction of buffer samples was consider basal sedimentation and subtracted from the other pellet fractions in each experiment. Data correspond to mean values of at least three independent experiments. Error bars correspond to standard deviations (*, P < 0.001). AspniGA: *Aspergillus niger* glucoamylase (positive control), ArathFt: *Arabidopsis thaliana* frataxin (negative control). OsttaSSIII-A D1 16 kDa, OsttaSSIII-A D2 15 kDa, OsttaSSIII-A D12 34 kDa, OsttaSSIII-B D1 8 kDa, OsttaSSIII-B D2 11 kDa, OsttaSSIII-B D 3 10 kDa, OsttaSSIII-B D123 48 kDa, ArathFt 15 kDa, and AspniGA 66 kDa.
10.1186/s13104-015-1598-6 Alignment between, ArathSSIII-CD, OsttaSSIII-CD, AgrtuGS CD and EsccoGS CD. Conserved positions, corresponding to glycogen binding sites in ArathSSIII CD, are shown in bold and shaded in grey. CD: catalytic domain; Arath: *Arabidopsis thaliana*; AgrtuGS: *Agrobacterium tumefaciens* glycogen synthase; Escco: *Escherichia coli* glycogen synthase.
