# Supplementary figures and images for: Functional demonstrations of starch binding domains present in Ostreococcus tauri starch synthases isoforms
Source: BMC Res Notes. 2015 Oct 28;8:613. doi: 10.1186/s13104-015-1598-6 (PMC4625611; doi:10.1186/s13104-015-1598-6)

**A.**

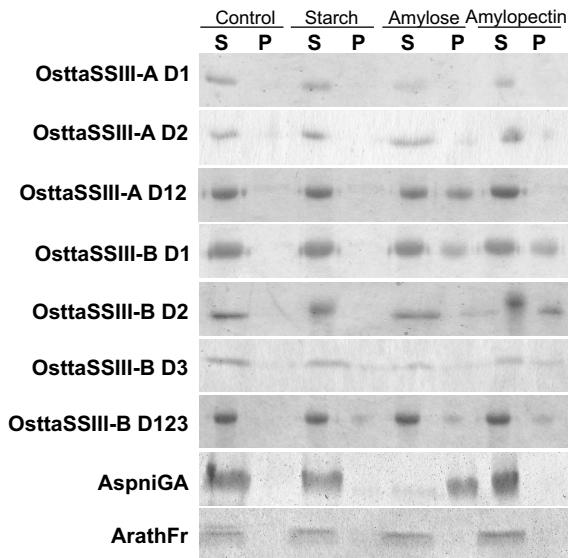

**B.**

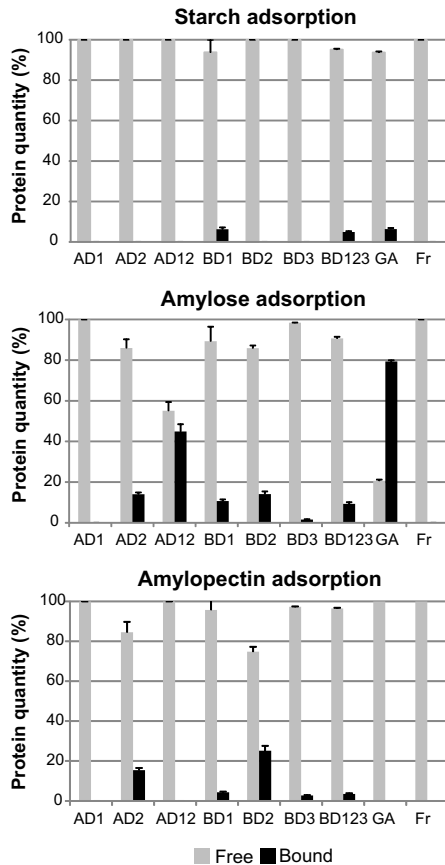

Supplement: Supplementary file 2 — 10.1186/s13104-015-1598-6 OsttaSSIII SBD-polysaccharide co-sedimentation assay. A. OsttaSSIIIs SBDs were incubated with starch, amylopectin, or amylose and examined for their ability to co-sediment. Then, they were localized to the supernatant (S) or pellet (P) fraction in SDS-PAGE, as described in Methods section. B. Densitometric quantifications of OsttaSSIII SBD-polysaccharide co-sedimentation assay presented in A. Polysaccharide bound or free protein levels are shown. The density for supernatant fraction of buffer incubated samples (control) was set to 100 % for each experiment, and density for pellet fraction of buffer samples was consider basal sedimentation and subtracted from the other pellet fractions in each experiment. Data correspond to mean values of at least three independent experiments. Error bars correspond to standard deviations (*, P < 0.001). AspniGA: Aspergillus niger glucoamylase (positive control), ArathFt: Arabidopsis thaliana frataxin (negative control). OsttaSSIII-A D1 16 kDa, OsttaSSIII-A D2 15 kDa, OsttaSSIII-A D12 34 kDa, OsttaSSIII-B D1 8 kDa, OsttaSSIII-B D2 11 kDa, OsttaSSIII-B D 3 10 kDa, OsttaSSIII-B D123 48 kDa, ArathFt 15 kDa, and AspniGA 66 kDa. [file 13104_2015_1598_MOESM2_ESM.pdf]
