# Supplementary material for: Functional demonstrations of starch binding domains present in Ostreococcus tauri starch synthases isoforms
Source: BMC Res Notes. 2015 Oct 28;8:613. doi: 10.1186/s13104-015-1598-6 (PMC4625611; doi:10.1186/s13104-015-1598-6)
Supplement: Supplementary file 3 — 10.1186/s13104-015-1598-6 Alignment between, ArathSSIII-CD, OsttaSSIII-CD, AgrtuGS CD and EsccoGS CD. Conserved positions, corresponding to glycogen binding sites in ArathSSIII CD, are shown in bold and shaded in grey. CD: catalytic domain; Arath: Arabidopsis thaliana; AgrtuGS: Agrobacterium tumefaciens glycogen synthase; Escco: Escherichia coli glycogen synthase. [file 13104_2015_1598_MOESM3_ESM.pdf]

```
ArathSSIII CD      574  EPPLHIVHIAVEMAPIAKVGGLGDVVTSLSRAVQELNHNVDIVFPKYDCIKHNFVKDLQ- 633
OsttaSSIIIACD      840  DKPLHVMSVSVEMAPIAKVGGLGDVVTSLGLAVQAEGHKVEVVLPKYDTLKYDLIEDLK- 910
OsstaSSIIIBCD      497  KPPLHVCHIAVEMAPIAKVGGLADVVTAIGRAIQDNGHLVEIILPKYQFFNNSVLLGGKE 556
OsttaSSIIIC        014  APILKVVHVAVEMAPIAKVGGMGDVVTALARATQEDGHQVEVFVPHYDIAQFENVDGYH- 072
AgrtuGS CD         001  ---MNVLSVSSEIYPLIKTGGLADVVGALPIALEAHGVRTRTLIPGYPAVKAAVTDPVKC 057
EsccoGS CD         001  ---MQVLHVCSEMFPLLKTGGLADVIGALPAAQIADGVDARVLLPAFPDIRRGVTD-AQV 056

ArathSSIII CD  634 FNRSYHWGGTEIKVWHGKVEGLSVYFLDPQNGLFQRGCVYG------CADDAGRFGFFCHAALEFLL--QGGFHP 700
OsttaSSIIIACD  911 EENGFHWGGCYNKVFSGTVEGVKTYFIDPENGMFRVGMIYGTDWLEIPMTDAERFGFFSRAALEWML--QSGRQP 971
OsstaSSIIIBCD  557 YETHFDWAGTTIRVEKCKVEGLQCFFIEPQNNMFQTDSVYG------RNDDAHRFNFFCNAALEFLV--RTARQP 623
OsttaSSIIIC    073 RAGEFKHEKTVVQVYKGWVEDVPVTLLRPENGFFDVGCIYG------RGDDHVRFDFFTDATLTWLR--SKQQEV 139
AgrtuGS CD     058 FEFTDLLG-EKADLLEVQHERLDLLILDAPAYYERSGGPYLGQTGKDYPDNWKRFAALSLAAARIGAGVLPGWRP 131
EsccoGS CD     057 VSRRDTFA-GHITLLFGHYNGVGIYLIDAPHLYDRPGSPYHDTNLFAYTDNVLRFALLGWVGAEMASGLDPFWRP 130

ArathSSIII CD  701 DILHCHDWSSAPVSWLFKD---------------------HYTQYGLIKTRIVFTIHNLEFG--ANAIGKAMTF  759
OsttaSSIIIACD  972 DIIHCHDWQTAPVAKAYWE---------------------DYHKYGLDNPRIVFTIHNLDFG--EGLVREAMDY 1022
OsstaSSIIIBCD  624 DILHCHDWSSAEVARAYWE---------------------HYHHNGLTKPKVAFTIHNMNYG--QAKLGEAVHH  674
OsttaSSIIIC    140 DVIHTHDWQTAAATWA-----------------------------GYPNAATALTVHNLQFG--VDRIRRGMES  182
AgrtuGS CD     132 DMVHAHDWQAAMTPVYMRYAETPEIPSLLTIHNIAFQGQFGANIFSKLALPAHAFGMEGIEYYNDVSFLKGGLQT  206
EsccoGS CD     131 DVVHAHDWHAGLAPAYLAARGRPAK-SVFTVHNLAYQGMFYAHHMNDIQLPWSFFNIHGLEFNGQISFLKAGLYY  204

ArathSSIII CD   760 ADKATTVSPTYAKEVAGNSVIS---------AHLYKFHGIINGIDPDIWDPYNDNFIPVPYTSENVVEGKRAAKE  826
OsttaSSIIIACD  1023 TQIGTTVSKTYAQEVSGHNSIS---------HQLEKFHGVVNGIDPDIWDPSDDKYLPVSFEMGSVVEGKAACRA 1088
OsstaSSIIIBCD   675 SQVATTVSPSYAGEVRGSPVIG---------NNGHKFTGVRNGIDPEIWDPETDIFVPVKYNADNQEEGKAAARA  740
OsttaSSIIIC     183 CDIATTVSPTYADEVRFHHAIA---------PSKDKFIGIRNGIDTDIWNPANDKFLPVGYNRSNAIDGKRAAAA  248
AgrtuGS CD      207 ATALSTVSPSYAEEILTAEFGMGLEGVIGS--RAHVLHGIVNGIDADVWNPATDHLIHDNYSAAN-LKNRALNKK  278
EsccoGS CD      205 ADHITAVSPTYAREITEPQFAYGMEGLLQQRHREGRLSGVLNGVDEKIWSPETDLLLASRYTRDT-LEDKAENKR  278

ArathSSIII CD   827 ELQNRLGLKS-ADFPVVGIITRLTHQKGIHLIKHAIWRTLERNGQVVLLGSAPDPRIQNDFVNLANQLHSSHG  898
OsttaSSIIIACD  1089 ALCARSNIPNRPDVPLVGVVTRLTHQKGIHLIKHAIYKAIERGCQVVLLGSAPDKKVQQEFEDMANHLKQSHF 1161
OsstaSSIIIBCD   741 ELRQRAGMTGWDDKPIVGVVSRLTAQKGVHLIKHAAHHTLSRGGQFVLLGSAPDPKIQGEFNGLANQLGG---  810
OsttaSSIIIC     249 ELCNRLGLEHPEGSPIVGVVSRLTAQKGIHLIKHACYRVLERGATFVLLGNAPDPAHQHDFNSLAKEMKEKYP  320
AgrtuGS CD      279 AVAEHFRID-DDGSPLFCVISRLTWQKGIDLMAEAVDEIVSLGGRLVVLG-AGDVALEGALLAAASRHHG---  345
EsccoGS CD      279 QLQIAMGLKVDDKVPLFAVVSRLTSQKGLDLVLEALPGLLEQGGQLALLG-AGDPVLQEGFLAAAAEYPG---  346
```
